# Vitamin D therapy in diabetic kidney disease

**Published:** 2014-01-01

**Authors:** Mahmoud Rafieian-Kopaei, Hamid Nasri

**Affiliations:** ^1^Medical Plants Research Center, Shahrekord University of Medical Sciences, Shahrekord, Iran; ^2^Department of Nephrology, Division of Nephropathology, Isfahan University of Medical Sciences, Isfahan, Iran

**Keywords:** Vitamin D, Diabetic nephropathy, Renal failure

**Implication for health policy/practice/research/medical education:**

Diabetic nephropathy is a major risk of end-stage kidney failure and is associated with greater morbidity and mortality, predominantly with augmented cardiovascular disease. Many complex factors relate to the progression of nephropathy of diabetic patients .Current investigations have focused on the optimization of renin-angiotensin system blockade in patients with diabetic nephropathy using combinations of drugs that target this pathway, however further studies have focused on the potential of new therapies that either target various pathways up-regulated by hyperglycemia or other targets believed to promote progression of diabetic nephropathy.

Recently an article published by Ahmadi *et al*, entitled “whether vitamin D3 is effective in reducing proteinuria in type 2 diabetic patients? (1). In a randomized double blinded parallel groups clinical trial, 51 diabetic individuals with proven diabetic kidney disease and vitamin D deficiency/insufficiency and stable hypertension, dyslipidemia, and hyperglycemic treatment were selected. Patients received oral vitamin D3 (pearl 50000 IU) or placebo one pearl every week for 12 weeks. Patients were assessed at baseline and 12 weeks after intervention from the point of 25(OH)D level, and urine albumin/creatinine ration (1). They assessed, plasma 25(OH)D level prior and after the intervention after and urine albumin/creatinine ratio. In their study, there was not a decrease in proteinuria in patients who obtained vitamin D for a period of 3 months (1). Diabetic nephropathy is a major risk of end-stage kidney failure and is associated with greater morbidity and mortality, predominantly with augmented cardiovascular disease (2,3). Many complex factors relate to the progression of diabetic nephropathy (4,5). Current investigations have focused on the optimization of renin-angiotensin system blockade in patients with nephropathy of diabetes using combinations of drugs that target this pathway (6-8), however further studies have focused on the potential of new therapies that either target various pathways up-regulated by hyperglycemia or other targets believed to promote progression of diabetic nephropathy for example the endothelin system, inflammation and vitamin D receptors (9-11). Up to now, various evidence supports, vitamin D as a negative regulator of the circulating and local tissue renin-angiotensin system, while the renin-angiotensin system have a critical role in the physiology of sodium and volume homeostasis (6-11). Excess activity of the renin-angiotensin system is associated with high blood pressure, kidney disease and diabetes. Indeed it is possible that, the most important putative mechanisms associating vitamin D to blood glucose control is its regulation of the renin-angiotensin system suppression of renin biosynthesis (2-8). It seems that treatment with vitamin D reduced the urinary urine albumin/creatinine ratio level by suppressing the compensatory renin increase in diabetic nephropathy (2-8). These beneficial consequences might be contributed to suppressed renal expression of renin and transforming growth factor beta (TGF-β) which may or may not be angiotensin II dependent (3-10). Recent studies have shown that low 25(OH)D3 levels are common in individuals with albuminuria. Thus, the anti-proteinuric efficacy of vitamin D in diabetic kidney disease is mediated by vitamin D receptor activation (4-11). More recent studies suggests that the effect of vitamin D receptor activation is partly that of negatively regulating renin-angiotensin system, which plays a significant role in the development of diabetic kidney disease (4-11). It is possible that, non-positive result on diminution of proteinuria by vitamin D therapy in the study of Ahmadi *et al*. may be due to short period of treatment. Also, in their study, it should be better to dissociate males from females and then analyze the data distinctly, while Houng *et al*. found that mean 25(OH)D3 concentrations were meaningfully lower in females than in males (12). In fact, a low vitamin D status was more closely associated with micro-albuminuria in males than in females (2-8,13). Limitations of study conducted by Ahmadi *et al*, need to be considered too. The sample size was small. In addition, limited duration of exposure to drug, as mentioned. Importantly, the season may affect vitamin D concentrations, and might also influence treatment outcome a factor that is very difficult to control. In addition, the best effective dose needs further exploration and finally, lack of evaluation of parathormone levels, while a bulk of evidences, revealed the positive association of parathyroid hormone and level blood pressure as an aggravating factor for diabetic kidney disease (2-9,12-14). Thus the negative result on reducing the proteinuria in diabetic kidney disease, should not discourage its use. We suggest, more prospective interventional studies with larger duration with control of confounders to better found this aspect of diabetic patients.

## Authors’ contributions

HN and MRK wrote the manuscript equally

## Conflict of interests

The authors declared no competing interests.

## Ethical considerations

Ethical issues (including plagiarism, informed consent, misconduct, double publication and redundancy) have been completely observed by the authors.

## Funding/Support

None declared.
